# Factors for successful vaginal delivery in twin pregnancies: a ten-year single-center retrospective study

**DOI:** 10.1186/s12884-025-08568-y

**Published:** 2025-12-08

**Authors:** Hiroki Ito, Takashi Shibata, Toshio Shimokawa, Hiroki Kato, Shigeki Nishikawa, Satoshi Nakago

**Affiliations:** 1 Department of Obstetrics and Gynecology, Takatsuki General Hospital, Takatsuki, Japan; 2 Department of Medical Data Science, Graduate School of Medicine, Wakayama Medical University, Wakayama, Japan

**Keywords:** Cesarean section, Mode of delivery, Predictive factors, Trial of labor, Twin pregnancy, Vaginal delivery

## Abstract

**Background:**

When certain conditions are met, a large randomized trial has suggested that for twin pregnancies, vaginal delivery does not increase adverse maternal or neonatal outcomes compared with cesarean section. However, the choice of the delivery mode is sometimes controversial, and policies can vary across different facilities. Identifying factors associated with successful vaginal delivery is important in reducing unnecessary cesarean sections.

**Methods:**

We retrospectively analyzed 370 twin pregnancies delivered after 32 weeks of gestation at our hospital between April 2012 and March 2022, excluding monoamniotic monochorionic twins. Vaginal delivery was offered when the first twin was in cephalic presentation, the estimated fetal weight of each twin was at least 1500 g, and there were no other indications for cesarean section. We compared maternal and fetal factors between successful and failed attempts at vaginal delivery. Notably, we used multivariable logistic regression and the classification and regression tree (CART) method for statistical analyses.

**Results:**

Of the 370 twin pregnancies, 133 women attempted vaginal delivery, and 115 (86.5%) achieved successful vaginal delivery of both twins. Multivariable analysis identified multiparity (adjusted odds ratio 15.29, 95% confidence interval 3.55–65.74; *p* < 0.001) and younger maternal age (adjusted odds ratio 0.84 per year, 95% confidence interval 0.73–0.96; *p* = 0.013) as independent predictors of successful vaginal delivery. CART analysis supported these findings, identifying parity as the primary discriminator. Among primiparas, maternal age ≤ 29 years emerged as a cutoff, with younger women having a higher probability of successful vaginal delivery. Univariable comparisons further supported this result.

**Conclusions:**

Our findings suggest that multiparous women, regardless of maternal age, and primiparous women aged ≤ 29 years are associated with a high probability of successful vaginal twin delivery.

## Background

For twin births, including both monochorionic diamniotic and dichorionic diamniotic pregnancies, vaginal delivery reportedly does not result in poorer outcomes for mothers or neonates compared with planned cesarean section, provided that appropriate conditions are met [1]. However, the optimal mode of delivery for twin pregnancies remains controversial [2], and policies differ between institutions. Even when a trial of vaginal delivery is medically feasible, a planned cesarean section is sometimes chosen based on the preferences of obstetricians or patients. Considering the maternal risks associated with cesarean delivery, reducing unnecessary procedures is an important issue in perinatal medicine [3].

Identifying factors that predict successful vaginal delivery and providing this information to patients may encourage more patients to attempt vaginal delivery and could ultimately assist in reducing unnecessary cesarean sections.

## Methods

### Study design and setting

To identify factors predictive of successful vaginal delivery in twin pregnancies, we conducted a retrospective cohort study at Takatsuki General Hospital, Osaka, Japan, between April 2012 and March 2022. The institutional ethics committee approved the study.

### Patients

Eligible cases were those of twin pregnancies delivered at ≥ 32 weeks of gestation. Exclusion criteria were stillbirth, single intrauterine fetal demise, selective reduction of multifetal pregnancy, and monoamniotic monochorionic twins. Patients who elected for planned cesarean section despite being eligible for vaginal delivery were excluded from the primary analysis.

### Permission criteria and management protocols for vaginal delivery

We offered patients the option of vaginal delivery when the first twin was in cephalic presentation and the estimated fetal weight of each twin was at least 1500 g, in accordance with previous studies [1]. Cases with indications for cesarean section or a history of prior cesarean delivery were excluded. We waited for the onset of spontaneous labor, and labor was induced when the Bishop’s score was ≥ 6 using continuous intravenous oxytocin. As an exception, in cases where expedited delivery was desirable, such as when there was fetal growth restriction or preeclampsia, oral prostaglandin E2 was administered for labor induction, even when the Bishop’s score was < 6. Cesarean section was performed when there was no spontaneous labor and a lack of cervical ripening by 39 weeks of gestation. Our facility does not use epidural anesthesia during labor. Trials of vaginal delivery were performed in the operating room in preparation for emergency cesarean section. Vaginal deliveries were attended by obstetricians with expertise in managing breech delivery of the second twin.

### Outcomes

The primary outcome was successful vaginal delivery of both twins. Failed attempts at vaginal delivery were defined as cesarean delivery for one or both twins during a trial of vaginal delivery, and this included cases in which the first twin was delivered vaginally but the second required cesarean delivery. Maternal and fetal factors compared between the successful and failed attempts were defined based on previous studies [2, 4]. Maternal factors included maternal age, short stature (≤ 150 cm), obesity (body mass index ≥ 25), parity, conception by assisted reproductive technology, hypertensive disorders, gestational diabetes mellitus, and induction of labor. Fetal factors included fetal growth restriction, presentation of the second twin, chorionicity (monochorionic diamniotic or dichorionic diamniotic), and inter-twin weight discordance (the second twin being ≥ 20% heavier than the first twin). Maternal and neonatal outcomes were also compared between the groups. The maternal outcome was defined as total fluid loss (blood and amniotic fluid) ≥ 2000 g. Neonatal outcomes were defined as Apgar score ≤ 7 at 1 or 5 min, or umbilical artery blood pH < 7.1 in either twin.

### Statistical analysis

Multivariable logistic regression was performed to identify independent predictors of successful vaginal delivery, with maternal age entered as a continuous variable (per year). Odds ratio (OR), adjusted odds ratio (aOR), and 95% confidence interval (CI) were used to quantify associations. A subgroup analysis was conducted for primiparas. In addition, we applied a classification and regression tree (CART) model to explore potential interactions [5] and to derive data-driven cutoffs for continuous predictors. CART recursively partitions the dataset to form subgroups with relatively homogeneous outcomes and automatically detects split points, accommodating both numerical and categorical predictors with intuitive visualization of decision rules. To avoid overfitting, the estimated CART model was pruned using 10-fold cross-validation. Leaves (terminal nodes) were summarized by the success rate with 95% CI. For the evaluation of subgroups obtained by the CART method, differences between nodes were summarized using ORs with 95% CI and compared using Fisher’s exact test. To validate CART-derived cutoffs, univariable comparisons using Fisher’s exact test were also performed. All statistical tests were two-sided, and *p* < 0.05 was considered statistically significant. Statistical analyses were performed using R version 4.2.1 (R Foundation for Statistical Computing, Vienna, Austria).

## Results

### Outcome of the delivery mode

**Fig. 1:**
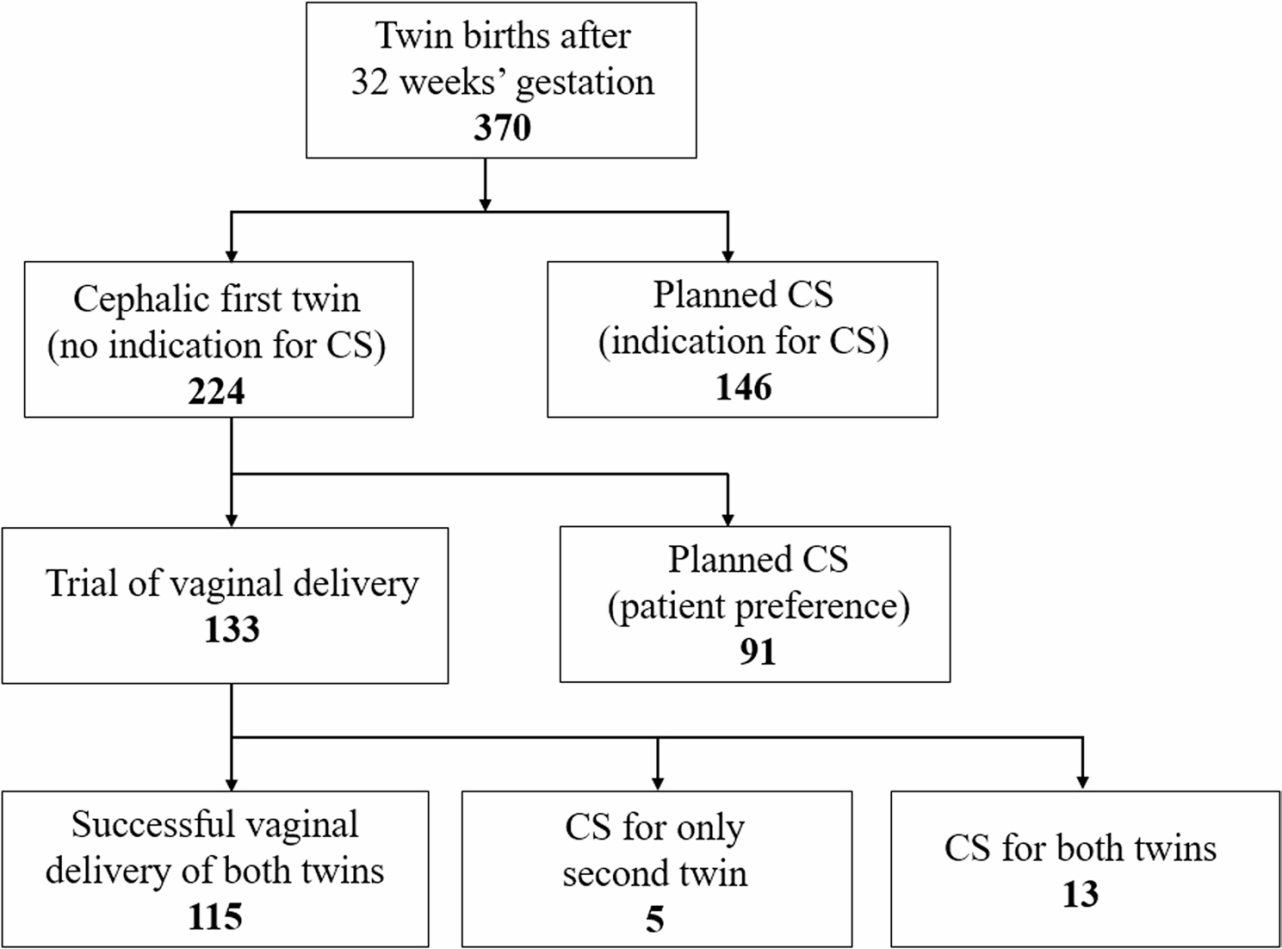
Flowchart of delivery mode. Of 370 twin pregnancies, 146 were not eligible for vaginal delivery due to clinical contraindications. Of the 224 eligible cases, 133 attempted vaginal delivery and 91 chose planned cesarean section. Among those who attempted vaginal delivery, 115 (86.5%) achieved vaginal delivery of both twins, whereas 18 (13.5%) required cesarean delivery. In 5 of these 18 cases, only the second twin required cesarean delivery. CS: cesarean section

Of 370 cases, 146 did not meet the criteria for vaginal delivery, and we performed cesarean sections (Fig. 1). Of the remaining 224 cases, vaginal delivery was attempted in 133 cases, while 91 women elected for planned cesarean section, despite meeting the criteria for vaginal delivery. Of the 133 patients who attempted vaginal delivery, 115 (86.5%) delivered both fetuses vaginally (successful vaginal delivery group), whereas 18 (13.5%) were converted to cesarean section during the attempt (failed attempts at vaginal delivery group). Indications for cesarean conversion were dystocia in 16 cases, non-reassuring fetal status in one case, and hypertensive disorder in one case. In five of the 18 failed attempts at vaginal delivery, only the second twin required cesarean delivery due to arrest of labor from malpresentation.

### Statistical analysis

**Fig. 2:**
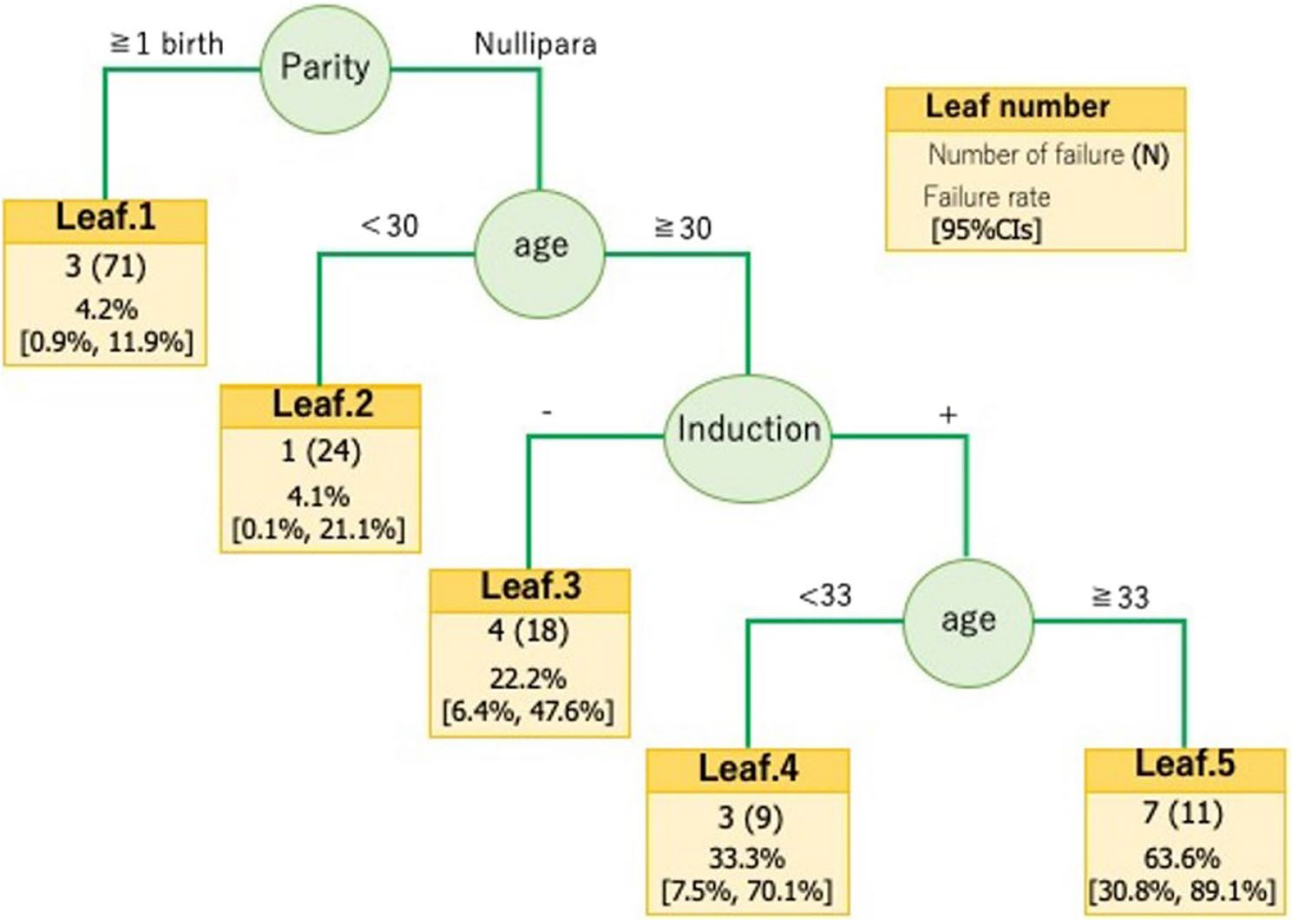
CART-derived success factors for vaginal delivery. Classification and regression tree (CART) model for identifying predictors of successful vaginal delivery. Parity was selected as the primary split. Among primiparas, maternal age ≤ 29 years was identified as the optimal cutoff. Leaf nodes display the number of failed cases (n), the total number of cases (N), the failure rate (%), and the 95% confidence intervals (CIs)

Multivariable logistic regression identified multiparity (aOR 15.29, 95% CI 3.55–65.74; *p* < 0.001) and maternal age (per year; aOR 0.84, 95% CI 0.73–0.96; *p* = 0.013) as independent predictors of successful vaginal delivery (Table 1). In the subgroup analysis of primiparas, younger maternal age was also associated with successful vaginal delivery (aOR 0.78, 95% CI 0.63–0.96; *p* = 0.020) (Table 2). CART analysis supported these findings: parity was the first split, and maternal age ≤ 29 years emerged as a key cutoff for primiparas (Fig. 2). These results were consistent with the logistic regression but provided a more intuitive and clinically applicable visualization. Univariable comparisons further supported the CART-derived cutoffs. Failed attempts at vaginal delivery among multiparous women were 4.2% (3/71), compared with 24.2% (15/62) among primiparas (OR 7.13, *p* < 0.001) (Table 3). Among primiparas ≤ 29 years of age, 4.2% (1/24) had failed attempts at vaginal delivery, compared with 36.8% (14/38) among primiparas ≥ 30 years of age (OR 13.40, *p* = 0.005) (Table 4). Multiparity and spontaneous conception were more common among women who attempted a trial of labor (53.4% vs. 33.0%, *p* = 0.003; 18.8% vs. 34.1%, *p* = 0.010), whereas other baseline characteristics were comparable between groups (Table 5).

**Table 1:** Multivariable logistic regression analysis of factors associated with successful vaginal delivery in twin pregnancies

	Successful vaginal delivery, *n*	Failed vaginal delivery, *n*	aOR	95% CI	*p* value
Multiparity (vs. primiparity)	68	3	15.29	3.55–65.74	< 0.001
Maternal age (per year)	-	-	0.84	0.73–0.96	0.013
Short stature (≤ 150 cm)	2	1	-	-	-
BMI ≥ 25	8	2	-	-	-
Conception by ART	19	6	-	-	-
Hypertensive disorders	9	2	-	-	-
Gestational diabetes	8	1	-	-	-
Induction of labor	96	15	1.20	0.27–5.27	0.807
Fetal growth restriction	12	2	-	-	-
Second twin non-cephalic	40	6	-	-	-
Intertwin weight discordance ≥ 20%	13	1	-	-	-

Numbers represent the exposed (“Yes”) category, with “No” as the reference (parity: primiparity as reference). Variables with low frequencies or non-convergence are presented as “-”

*aOR* adjusted odds ratio, *CI* confidence interval, *BMI* body mass index, *ART* assisted reproductive technology

**Table 2:** Multivariable logistic regression analysis of factors associated with successful vaginal delivery in primiparas

	aOR	95% CI	*p* value
Maternal age (per year)	0.78	0.63–0.96	0.020

Multivariable logistic regression in primiparas including maternal age, adjusted for induction of labor, BMI ≥ 25, ART, hypertensive disorders, gestational diabetes, fetal growth restriction, second-twin non-cephalic presentation, intertwin weight discordance ≥ 20% (second twin heavier), and short stature (≤ 150 cm)

*aOR* adjusted odds ratio, *CI* confidence interval

**Table 3:** Univariable comparison of failed attempts at vaginal delivery between multiparas and primiparas

	Successful, *n*	Failed, *n*	OR (95% CI)	*p* value
Multipara	68 (95.8%)	3 (4.2%)	7.13 (2.00–25.3)	< 0.001
Primipara	47 (75.8%)	15 (24.2%)	1.00 (Reference)	-

Fisher’s exact test was used for univariable analysis

*OR* odds ratio, *CI* confidence interval

**Table 4:** Univariable comparison of failed attempts at vaginal delivery between primiparas aged ≤ 29 and ≥ 30 years

Maternal age (primiparas)	Successful, *n*	Failed, *n*	OR (95% CI)	*p* value
≥ 30 y	24 (63.2%)	14 (36.8%)	1.00 (Reference)	-
≤ 29 y	23 (95.8%)	1 (4.2%)	13.40 (1.6–115)	0.005

Fisher’s exact test was used for univariable analysis

*OR* odds ratio, *CI* confidence interval

**Table 5:** Baseline characteristics of women who attempted vaginal delivery versus those who elected Cesarean section despite eligibility for vaginal delivery

	Attempted vaginal delivery (*n* = 133)	Elected cesarean section (*n* = 91)	*p* value
Maternal age (years)	32.26 ± 4.94	32.85 ± 5.04	0.386
Parity (multipara, %)	53.4	33.0	0.003
BMI ≥ 25 (%)	7.5	9.9	0.532
Conception by ART (%)	18.8	34.1	0.010
Monochorionic diamniotic (%)	34.6	30.8	0.551
Second twin non-cephalic (%)	34.6	30.8	0.551
Weight discordance ≥ 20 (%)	10.5	9.9	0.878
Hypertensive disorders (%)	8.3	8.8	0.891
Gestational diabetes (%)	6.8	9.9	0.398
Short stature ≤ 150 cm (%)	2.3	8.8	0.054

Values are presented as mean ± SD or %. *p* values are shown for group comparisons. The Welch t test was used for continuous variables, and the chi-square test or Fisher’s exact test for categorical variables

### Maternal and fetal outcomes

Maternal and neonatal outcomes are summarized in Table 6. Total fluid loss ≥ 2000 g occurred in seven of the 115 cases in the successful vaginal delivery group and in two of 18 cases in the failed attempts at vaginal delivery, without statistical significance. The incidence of Apgar score ≤ 7 at 1 or 5 min showed no significant differences between groups. However, umbilical artery pH < 7.1 was more frequent in the second twin of the failed attempts at vaginal delivery compared with those in the successful vaginal delivery group (0/115 vs. 2/18, *p* = 0.019).

**Table 6:** Maternal and neonatal outcomes between successful and failed attempts at vaginal delivery

	Successful, *n*	Failed, *n*	*p* value
Maternal total fluid loss ≥ 2000 g	7 (6.1%)	2 (11.1%)	0.615
Apgar score ≤ 7 at 1 min (first twin)	12 (10.4%)	4 (22.2%)	0.244
Apgar score ≤ 7 at 1 min (second twin)	38 (33.0%)	6 (33.3%)	1.000
Apgar score ≤ 7 at 5 min (first twin)	2 (1.7%)	1 (5.6%)	0.370
Apgar score ≤ 7 at 5 min (second twin)	3 (2.6%)	2 (11.1%)	0.147
Umbilical artery pH < 7.1 (first twin)	0 (0%)	0 (0%)	1.000
Umbilical artery pH < 7.1 (second twin)	0 (0%)	2 (11.1%)	0.019

Fisher’s exact test was used for univariable comparisons

## Discussion

The success rate of vaginal twin delivery in our institution was 86%, which is consistent with previous reports [2, 4, 6–8]. In the multivariable analysis, multiparity and younger maternal age were identified as independent predictors of successful vaginal delivery, which is in line with previous studies that reported higher success rates in multiparous women [2, 6, 9]. Approximately one quarter of primiparas failed to deliver vaginally, which prompted a separate analysis of this group. Among primiparas, maternal age remained a significant predictor. CART analysis identified 29 years as the data-derived cutoff, which differs from the conventional threshold of advanced maternal age (≥ 35 years) [9]. This cutoff was supported by univariable comparisons, which showed a significantly higher success rate in primiparas ≤ 29 years old than in those ≥ 30 years old. A possible explanation for the lower success rate at higher maternal ages is a decline in uterine contractile function, along with a reduced oxytocin receptor density, both of which have been associated with labor dystocia in previous studies [10, 11].

In this study, approximately 40% of patients who were eligible for vaginal delivery nonetheless opted for planned cesarean section despite the absence of specific indications. We speculate that their decision was influenced by vague apprehension about the possibility of emergency conversion to cesarean delivery and perceived fetal risks, including unforeseen intrapartum complications or adverse outcomes reported in other cases [12, 13]. Among these women, 58.7% were either primiparas ≤ 29 years old or multiparas. Providing individualized information based on our findings may support shared decision-making and encourage these patients to attempt vaginal delivery, thereby helping to reduce unnecessary cesarean deliveries [4].

In our analysis of neonatal outcomes, Apgar scores did not significantly differ between the first and second twins. Meanwhile, although the overall number of cases was small, umbilical artery pH < 7.1 occurred more frequently in the second twin when there were failed attempts at vaginal delivery. This finding is consistent with previous studies, and it suggests that the second twin may be more likely to be exposed to intrapartum hypoxia [12]. When the inter-delivery interval between twins is prolonged, the second twin has been reported to show lower Apgar scores and umbilical artery pH [13]. Based on our clinical experience, this may be due to intrauterine changes following the delivery of the first twin, including fetal malpresentation, altered placental perfusion, placental abruption, or cord compression, which can lead to transient hypoxia in the second twin. Although such risks exist, planned cesarean delivery does not appear to reduce them, and maternal and neonatal outcomes have not been shown to differ significantly between planned cesarean and vaginal delivery [1]. In addition, as observed in our cases, these intrauterine factors have also been reported not to necessarily result in increased neonatal morbidity [13]. Importantly, as a prerequisite, twin delivery should be managed by experienced providers prepared for breech delivery of the second twin [14]. Limitations of this study include its retrospective single-center design, the exclusion of patients who opted for planned cesarean section despite eligibility, the absence of epidural analgesia, the non-implementation of vaginal birth after cesarean, and the relatively small sample sizes in some subgroups, which may affect generalizability. The findings require confirmation in prospective studies or interventional research.

## Conclusion

Our results indicate higher success rates of vaginal delivery in twin pregnancies among multiparas, regardless of age, and among primiparas aged ≤ 29 years.

## Data Availability

All data generated or analyzed during this study are included in this published article.

## Abbreviations

aOR: Adjusted odds ratio
ART: Assisted reproductive technology
BMI: Body mass index
CART: Classification and regression tree
CI: Confidence interval
OR: Odds ratio
